# Multiscale Structural Engineering of Cellulose Foams: Performance Characterization and Fiber Imaging

**DOI:** 10.3390/polym17172355

**Published:** 2025-08-29

**Authors:** Patricija Pevec, Urška Kavčič, Aleš Hladnik, Diana Gregor-Svetec

**Affiliations:** 1 Pulp and Paper Institute, Bogišićeva 8, 1000 Ljubljana, Slovenia; urska.kavcic@icp-lj.si; 2 Faculty of Natural Sciences and Engineering, University of Ljubljana, Aškerčeva cesta 12, 1000 Ljubljana, Slovenia; ales.hladnik@ntf.uni-lj.si

**Keywords:** fiber foams, cellulose foams, sustainable materials, bio-based materials, PCA, protective packaging

## Abstract

The paper industry is always looking for possible solutions for new fiber-based products, such as protective and cushioning materials. These materials must be carefully designed to provide effective cushioning while also being lightweight to reduce transportation costs. Additionally, they need to offer protection from environmental and mechanical damage, besides having good processability to ensure proper buffering. The widely used protective and cushioning materials, such as plastic foams and expanded or extruded polystyrene, create significant disposal challenges. Therefore, there is increasing demand for biodegradable and sustainable materials for cushioning applications. The focus of our research was to develop fiber-based foams and investigate the influence of different compositions (hardwood and softwood) of cellulose fibers on the basic (mass, thickness, density) and mechanical properties (three-point bend test, tensile properties). Foams made entirely from short eucalyptus fibers (100S) exhibited the highest density (28.0 ± 0.34 kg/m^3^) and lowest thickness (38.82 ± 4.21 mm), resulting in superior tensile strength and elastic modulus but lower strain at break. In contrast, foams composed of long spruce fibers (100L) had the lowest density (19.0 ± 0.27 kg/m^3^) and highest thickness (58.52 ± 1.50 mm), with lower strength and stiffness but much higher ductility and porosity (confirmed by ~30% higher air permeability compared to 100S). Blended formulations demonstrated intermediate behavior, with the 50S50L foam showing a favorable balance of strength, stiffness, and flexibility. Visual analysis confirmed heterogeneous fiber distribution with localized agglomerates and compaction at the bottom layer due to casting. To further interpret the complex relationships within the dataset and uncover patterns, Principal Component Analysis (PCA) was applied to all experimental results. The findings of the research contribute to the broader understanding of how different fiber types and blends impact the performance of sustainable cellulose-based foams, with potential implications for the development of biodegradable packaging and lightweight construction materials.

## 1. Introduction

The protective and cushioning materials are crucial in packaging, especially for fragile goods like electronics and glass. These materials must be carefully designed to provide effective cushioning while also being lightweight to reduce transportation costs. Additionally, they need to protect the products from mechanical damage and offer barrier properties to prevent moisture damage besides having good processability to ensure proper buffering. The widely used protective and cushioning materials, such as plastic foams and expanded or extruded polystyrene, create significant disposal challenges. These materials are lightweight and bulky, making them difficult to recycle economically and environmentally due to high handling and transportation costs [1]. Additionally, they are not biodegradable and are hard to dispose of through soil or composting, contributing to environmental concerns [2]. The number of packages shipped globally is growing rapidly and is expected to increase significantly in the next few years. A small percentage of plastics is reused or recycled, and 80% is either landfilled, incinerated, or leaked into the environment [3]. There is an urgent need to replace petroleum-based plastic packaging, developed during the last century for transport and protection needs, with sustainable alternatives, focusing significantly on bio-based foams. These materials have gained attention as eco-friendly substitutes for traditional foams like expanded polystyrene (EPS) and expanded polyethylene (EPE), which are non-biodegradable and environmentally detrimental [4]. Governments worldwide are enacting stricter regulations to reduce plastic waste, creating a higher demand for bio-based raw material sources such as cellulose-based materials. Cellulose is a natural, bio-based polymer that is abundant, biodegradable, and recyclable, making it an attractive option for packaging applications. However, while cellulose-based materials have clear environmental benefits, the transition to fiber-based materials faces significant technical and economic challenges.

For industries aiming to become more resource-efficient, the ultimate challenge is achieving material reductions without diminishing the performance standards expected of the end product [5]. In this case, the foam-forming process offers several advantages compared to conventional wet-forming methods, such as reduced water usage and improved fiber distribution. It enables the production of a variety of fiber-based products, including paper, lightweight packaging, nonwoven fabrics, filters, and insulation materials [2]. Furthermore, foam forming allows for the development of novel applications featuring precise layering of materials, complex three-dimensional (3D) shapes, and customized microstructures [6]. In the early 1970s, the possibility of replacing water with foam in papermaking was investigated by several researchers [7,8,9], leading to the development of the Radfoam process [5,10]. Anyway, foam forming was not adopted in the paper industry at that time, despite its potential, and in 2013, the idea of using wet foams as a carrier phase to replace water has again captured interest [5]. The foam-forming process for producing cellulose materials has been extensively developed in Finland at the VTT Research Center, and since 2013, the world’s first pilot plant for foam-formed cellulose materials has been in operation [2]. In 2021, Stora Enso introduced Papira^®^, a lightweight and shock-absorbing material made from wood pulp foam, for consumers [11]. This material is a promising alternative for protective and cushioning packaging [2].

Aqueous foams are categorized into three groups based on their air content: bubbly liquids, wet foams, and dry foams. Fiber-foam at VTT, Finland, is generated by introducing air into a water-fiber suspension with the help of surfactants that reduce the surface tension between water and air, stabilizing bubbles. Since fibers are hygroscopic and hydrophilic, water can swell and soften them, allowing mechanical entanglement and short-range (<nm) interactions to tightly bind the fibers. This process produces a strong yet lightweight, sustainable, and biodegradable material [6].

Usually, for foam forming, the required foam suspension is generated by the mechanical mixing of water, a foaming agent, and cellulose fibers. There are a few other different methods to produce aqueous foam, such as blowing gas through a nozzle into a liquid, producing foam through gas nucleation, shaking or blending, or sparging gas through a porous membrane. Foaming agent sodium dodecyl sulfate (SDS) is advantageous because its low molecular weight allows for high air content at low concentrations. However, a downside of anionic surfactants like SDS is their sensitivity to water hardness, which can be mitigated by blending SDS with other surfactants [6].

Air content in foam is a key quality indicator, although other foam properties, such as bubble size and half-life, are also very important foam properties. At the start of mixing, a vortex typically forms at the center of the vessel. Mixing is generally continued until the desired air content (around 50–70%) is reached or until the vortex closes. Once the fiber foam suspension is ready, mixing is halted and the wet fiber foam is transferred into a modified handsheet mold. To help orient the fibers along the flow direction, foam is often decanted into the mold using a tilted plate. If the foam were poured directly into the center of the mold from a container, it would spread radially along the fabric, creating circular patterns in the formed sheet. Density variations in the structure can be achieved by initially applying a brief vacuum pulse, followed by allowing gravity to drain the remaining free water from the sample. This process results in a denser structure [6].

In the field of fiber foams, different studies were performed. Research by Al-Qararah et al. showed that in the deposition process, the suspending medium has a significant role in determining the structural properties [12]. The clear difference is in the microscopic pore structures of the foam-formed and water-formed sheets. But the most essential factor that controls the properties of the resulting fiber networks is the fiber type [12]. Ketola et al. investigated foam-formed materials using different combinations of hydrophilic and hydrophobic viscose fibers with foaming agents [13]. The study highlighted that both the type of viscose fiber and the choice of foaming agent affect foam quality and the performance of the final material [13]. Alimadadi and Uesaka produced foam by mixing refined TMP reject from a Swedish paper mill and sodium dodecyl sulfate (SDS) set to 0.4 g/L at rotational speeds between 500 and 2000 rpm, then transferred it to a wire at a low speed of 0.1 m/s [14]. To reduce in-plane fiber orientation, they carefully controlled water drainage and foam collapse by drying the foam slowly at 70 °C for at least 3 h. Their findings emphasized that slow and controlled drying is essential to maintain a random fiber orientation and ensure uniform structural properties in foam-formed materials [14]. Lu et al. developed a novel class of lignocellulose-based foams using a soft-template method with the amphiphilic polymer Pluronic F127 [15]. The resulting foams exhibited a low density (0.055 g/cm^3^), high compressive strength (0.25 MPa at 80% strain), and strong recoverability. These foams also significantly outperformed conventional molded pulp and EPE in cushioning and thermal insulation tests. The truss-like porous microstructure, created by fiber-PVA-F127 interactions and stabilized through hydrogen bonding, proved critical in enabling both structural integrity and resilience. Freville et al. reviewed recent technological advances in producing three-dimensional (3D) cellulosic materials for packaging [4]. The review categorizes emerging fabrication techniques such as dry-molded cellulose, cellulose injection molding, foam forming, and cellulose 3D printing. These methods aim to bridge the performance gap between cellulose-based and petroleum-derived packaging, especially for applications requiring complex geometries and durability. The authors highlight that while molded pulp is well-established, novel methods face challenges including reproducibility, mechanical performance optimization, and scalability. Their analysis reveals that process engineering and raw material heterogeneity remain major hurdles and that industrial adoption of 3D cellulosic packaging still lacks strong scientific backing despite growing commercial interest [4]. The study of Zhang et al. explored a new way to produce cellulose–paraffin composite foams designed for thermal energy regulation. The researchers used a one-step emulsion–gelation method in which molten paraffin was dispersed in a cellulose/LiBr aqueous solution to create an oil-in-water emulsion. Upon cooling, the mixture solidified into a gel, forming a three-dimensional network of cellulose nanofibrils with spherical paraffin particles (1–8 μm). No chemical bonding occurred between the cellulose and paraffin, but the structure effectively trapped the paraffin and prevented leakage. The resulting foams showed notable properties: high mechanical strength, with a maximum elastic modulus of 7.08 MPa, high latent heat storage and low thermal conductivity. This research builds upon the structural and insulating qualities of traditional cellulose foam but enhances its functionality by incorporating paraffin as a phase change material [16].

The goal of our research was to develop the bio-based foams made from cellulose fibers and to determine the influence of different fiber compositions in foams on their basic and mechanical properties.

## 2. Materials and Methods

### 2.1. Cellulose Fibers

For the production of cellulose-based fiber foams, two different wood species were used: deciduous and coniferous. Cellulose sheets of the aforementioned fibers were used for the preparation of fiber foams, dry mass was determined, and five different samples with different ratios of short (Eucalyptus bleached kraft pulp, BEKP, Santa Fe, Santiago, Chile) and long (ECF-bleached softwood sulfate pulp ORION sheet, Heinzel Pöls, Styria, Austria) fibers were prepared as presented in Table 1.

The fibers used were analyzed regarding morphological properties. Homogenous water suspension was prepared and Valmet Fiber Image Analyzer (Valmet FS5, Valmet, Espoo, Finland) was used to determine the fiber length (ISO 16065-2 [17]), diameter, fines, curl, kink and fibrillation.

### 2.2. Fiber Foams Preparation

#### 2.2.1. Foam Forming

**Figure 1:**
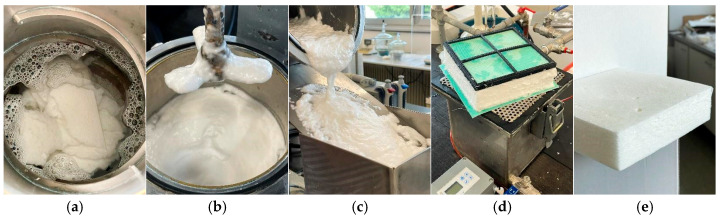
Foam forming process: (**a**) prepared surfactant solution with fibers, SDS and water; (**b**) solution after 10 min of mechanical stirring; (**c**) pouring the foam into the foam forming machine; (**d**) foam with frame and filter after water draining; (**e**) dry foam.

The foam samples’ preparation began by preparing a surfactant solution by dissolving 1 g of sodium dodecyl sulfate (SDS) in 1 L of water. This surfactant-rich solution enables the formation of a stable foam by lowering the surface tension of water. Once the SDS was dissolved, 30 g (dry mass) of cellulose fibers in varying ratios (Table 1) were gradually added to the solution (Figure 1a). The solution was then subjected to vigorous mechanical stirring in a laboratory disintegrator, which incorporates air and generates a uniform foam structure. The disintegrator rotation speed was set to 3000 revolutions per minute (RPM), and the duration of mixing was always set to 10 min to ensure consistency with all the samples. Within this foam, air bubbles are stabilized by the surfactant and become physically embedded between the cellulose fibers, effectively preventing fiber flocculation and promoting uniform dispersion (Figure 1b). The quantities of water and SDS were kept constant in all formulations to isolate and assess the effects of fiber composition on the properties of the resulting dry foams.

The foam-fiber suspension was then subsequently transferred into a foam-forming machine made at the Pulp and Paper Institute. A foam-forming machine has a perforated plate, where it is allowed for the foam to drain under gravity and light vacuum to speed up the dewatering process without causing substantial compression. After the liquid solution was poured into the machine down the inclined metal plate (that kept the foam flow constant) (Figure 1c), the premade 3D frame with inserted filter made for paper machines was put on top of it to keep the foam straight and steady. Under the influence of fiber suspension flowing down the inclined metal plate, the fibers in foam slightly aligned in two directions machine direction (MD) and cross direction (CD). To conserve energy, it is important to remove as much water as possible from the fiber network through drainage before drying in any kind of oven. As the foam collapses a little and water is in the most part removed, the fibers remain evenly distributed, forming a wet foam with a highly open and lightweight structure (Figure 1d). Draining lasted 5 min. Foam, together with the frame and filter, was then considered ready for drying.

#### 2.2.2. Drying

Prepared foam samples were dried in the air ventilator oven dryer at 65 °C for 5 h to evaporate the rest of the water. After drying in the oven dryer, the material stayed in frames and filters at room temperature for another 15 h. The result is a highly porous fibrous material with a thickness of 38–59 mm (Figure 1e).

#### 2.2.3. Sample Preparation

To ensure accurate and reproducible sample preparation, the foam specimens were cut to the appropriate sample’s dimensions using a precision Metabo miter saw (Metabo KGS 305 M, Metabo, Nürtingen, Germany) operating at a constant blade rotation speed of 3700 RPM. The high-speed cutting allowed for clean and uniform cross-sections without inducing significant deformation, thermal damage, or compression of the lightweight and porous cellular structure. The use of a fine-toothed blade further minimized surface irregularities and edge artifacts, which is particularly critical when preparing samples for structural, mechanical, or microscopic analyses. All cutting procedures were carried out under controlled laboratory conditions to maintain consistency and to prevent contamination or damage to the foam microstructure.

### 2.3. Analyses of the Foam Samples

All the produced foam samples were analyzed regarding thickness, density, and air permeability. Sample foams were first cut 15 cm × 15 cm to measure their mass. Thickness was measured on different spots of foam samples using calipers and air permeability was determined using the Frank PTI Bendtsen tester according to modified ISO 5636-3:2013 [18] on samples cut to 7.5 cm × 7.5 cm. Besides basic properties, the three-point bend test was measured on samples sized 4 cm × 15 cm, according to adapted ISO 5628:2019 [19] while tensile properties were measured according to ISO 1798:2008 [20] (sample size was 1 cm × 15 cm). Both tests were measured on Zwick Roell Z010 (Class 0.5, ISO 7500-1 [21]). Before the testing, all the samples were conditioned for at least 24 h at 23 °C and 50% relative humidity according to ISO 187 standard [22].

Additionally, the porous network was examined by confocal microscopy imaging (Leica TCS SP2).

## 3. Results

### 3.1. Morphological Properties of Cellulose Fibers

In fiber-based materials, the combination of different fiber types could be used to optimize the balance between strength and formation, which is essential in foam-forming applications where lightweight and well-formed samples are needed. Morphological properties of the fibers used for the sample’s preparation are presented in Table 2.

### 3.2. Basic Properties of Fiber Foams

All five samples of cellulose foams were tested for basic properties, i.e., mass, thickness, and density. As can be seen from Table 3, the mass of foams is similar; the difference is less than 2.5%, whereas other characteristics of the cellulose foams are more influenced by the fiber composition. In contrast, both thickness and density showed marked variation depending on the short (S) to long (L) fiber ratio.

### 3.3. Mechanical Properties of Fiber Foams

**Figure 2:**
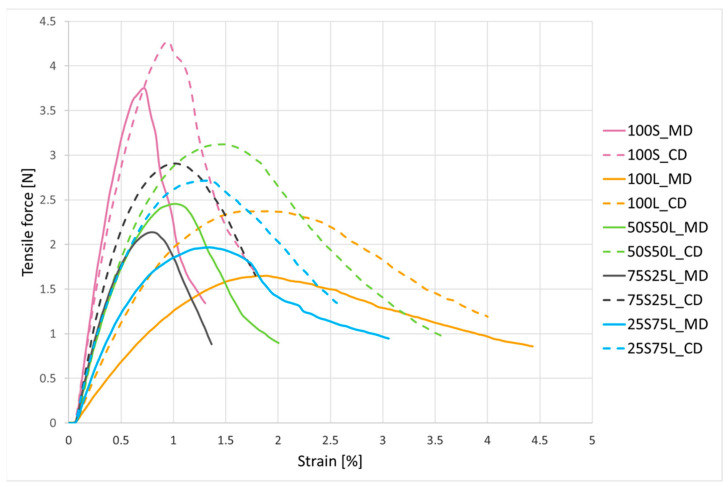
Tensile force/strain curves of fiber foams.

After cellulose fiber foams were tested for tensile and bending properties. The continuous record of increasing tensile force acting on fiber foam versus strain was recorded. Obtained curves are presented in Figure 2. The test was performed on samples cut in two directions; in the direction of fibers flowing down the inclined metal plate, marked as machine direction (MD), and in perpendicular direction, marked as cross direction (CD). Some of the tensile and bending properties determined are presented in Table 4.

### 3.4. Visual Analysis of Fiber Foams

**Figure 3:**
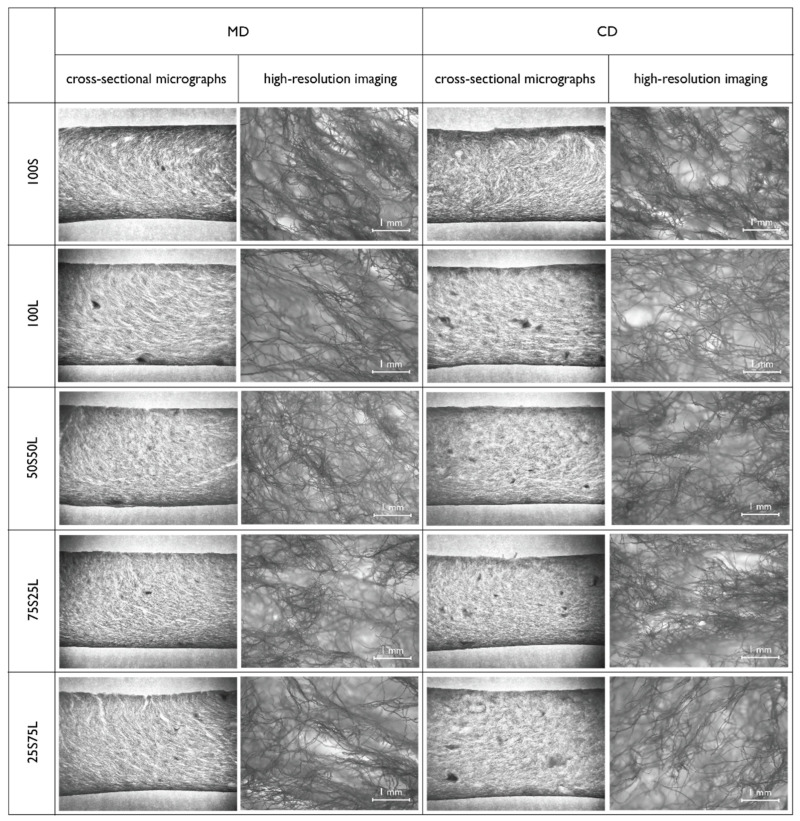
Cross-sectional micrographs and high-resolution imaging of fiber foams.

Microscopic characterization of the cellulose foams was conducted to examine the internal fiber morphology and structural organization (Figure 3). Cross-sectional micrographs were obtained to visualize the spatial arrangement and connectivity of the fibers within the foam matrix. Furthermore, high-resolution imaging enabled detailed observation of fiber distribution patterns in relation to the compositional heterogeneity of the foam. Visual analysis was systematically performed across all sample types, accounting for both machine direction (MD) and cross direction (CD) fiber orientations, thereby allowing for a comprehensive assessment of anisotropy and structural variation within the materials.

### 3.5. PCA of the Results

**Figure 4:**
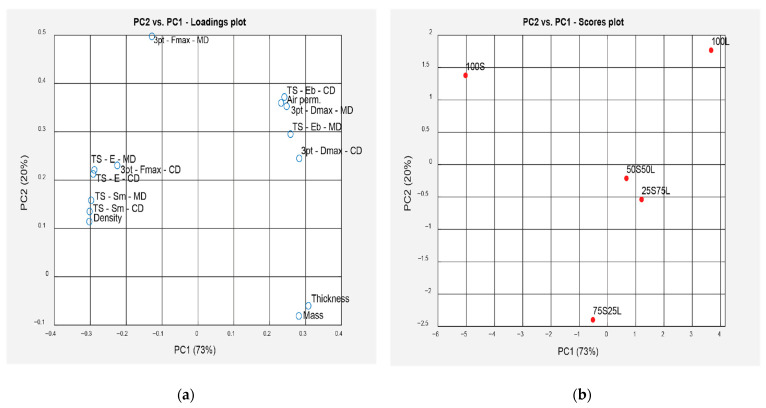
(**a**) The position of the standardized original variables (loadings plot) where 3pt-Fmax-MD/3pt-Fmax-CD: three-point bend test maximum force in machine and cross direction, 3pt-Dmax-MD/3pt-Dmax-CD: three-point bend test maximum flexure in machine and cross direction, TS-Sm-MD/TS-Sm-CD: tensile test maximum force in machine and cross direction, TS-Eb-MD/TS-Eb-CD: tensile test strain at break in machine and cross direction, TS-E-MD/TS-E-CD: tensile test elastic modulus in machine and cross direction, Mass, Thickness, Density and Air permeability, (**b**) the location of all samples (scores plot) in the PC1–PC2 space; (**b**) the location of the samples (scores plot) in the PC1–PC2 space.

PCA served as a powerful multivariate statistical tool that enabled dimensionality reduction while preserving the most significant variance in the data. This comprehensive PCA-based analysis provided deeper insight into the interdependence between fiber ratio and resulting foam properties, strengthening the robustness of our conclusions (Figure 4).

Our research had a relatively simple system comprising only five samples and fourteen measured variables or parameters. Principal Component Analysis (PCA) transforms the original variables into an equal number of new variables—principal components (PCs)—which are mutually independent (orthogonal).

**Figure 5:**
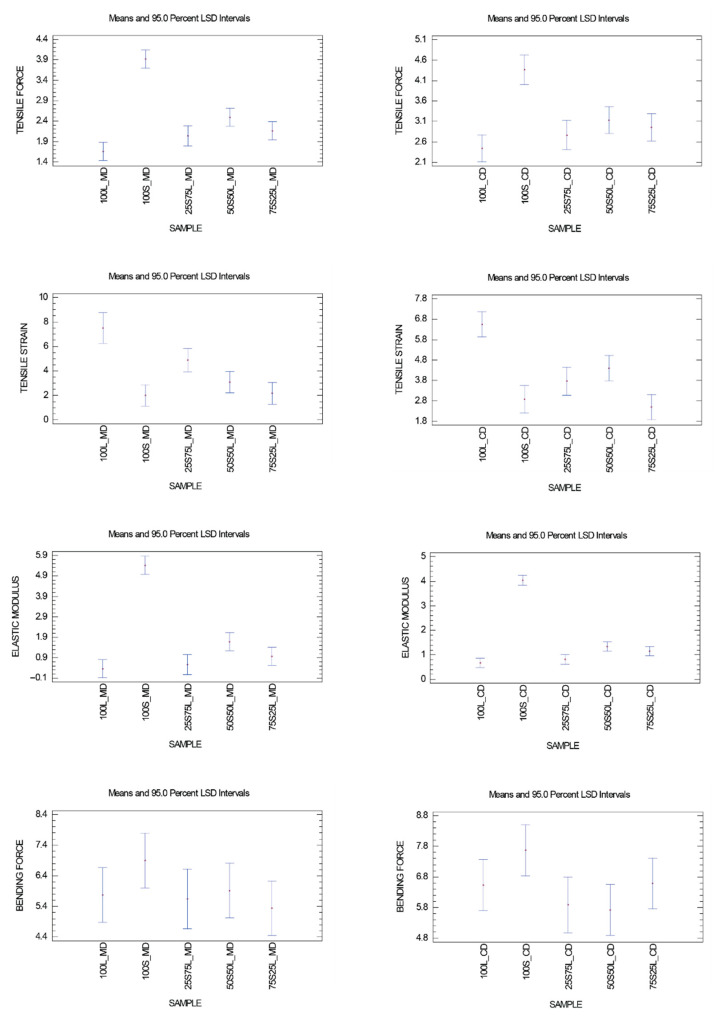
Mean values with 95% Least Significant Difference (LSD) confidence intervals for tensile force, tensile strain, elastic modulus, and bending force in MD and CD.

A series of one-way ANOVA tests were conducted for each of the four mechanical properties in both the MD and CD. The analyses revealed statistically significant differences among the sample means for tensile force, tensile strain, and elastic modulus at the 95% confidence level (Figure 5). In contrast, no significant differences were detected for bending force.

## 4. Discussion

Results from Table 2 confirm that the spruce fibers are significantly longer than eucalyptus fibers. Longer fibers generally contribute more to mechanical strength, while shorter fibers improve uniformity and surface smoothness. The spruce fibers are also thicker, which usually results in fibers. Longer fibers generally contribute more to mechanical strength, while shorter fibers improve uniformity and surface smoothness. The spruce fibers are also thicker, which usually results in a bulkier structure with higher porosity, while thinner eucalyptus short fibers may lead to a denser structure. In foam forming, a balance is needed to achieve both good strength and formation quality. Both fiber types show moderate curl. A slightly higher curl in long fibers may contribute to better entanglement and web strength but can also reduce bonding potential. A higher kink index in spruce fibers suggests more irregular shape or mechanical deformation, possibly from refining. Kinked fibers may decrease bonding potential due to poor contact area but can improve formation bulk and flexibility. Fibrillation is low in both cases. Slightly higher fibrillation in spruce fibers may enhance bonding but is still minimal overall. For stronger bonding, a higher degree of fibrillation is desirable. Long spruce fibers have higher total fines, resulting from broken fragments usually connected to refining. High fines improve formation but can reduce drainage and increase binder demand. Excessive fines may also negatively impact foam strength.

The foam composed solely of short fibers (100S) exhibited the lowest thickness (38.82 ± 4.21 mm) and the highest density (28.0 ± 0.34 kg/m^3^), suggesting a more compact, less porous structure. In contrast, the 100L sample, composed entirely of long fibers, showed the greatest expansion, with the highest thickness (58.52 ± 1.50 mm) and the lowest density (19.0 ± 0.27 kg/m^3^). These differences can be attributed to the thicker fibers, higher quantity of fines, enhanced fiber entanglement, and network formation afforded by the longer fibers, which promote a more open and voluminous foam structure. Measurements of air permeability confirm a more porous, bulkier structure in the case of the foam composed solely or mainly of long fibers. The difference between the air permeability of both foams made from solely short or long fibers is almost 30%.

Blended formulations demonstrated intermediate behavior. The 50S50L and 25S75L samples exhibited relatively low densities (20.3 ± 0.28 and 19.6 ± 0.21 kg/m^3^) and high thicknesses (54.43 ± 3.04 and 55.97 ± 3.75 mm), closely resembling the properties of the 100L foam. In contrast, the 75S25L sample displayed a higher density (21.9 ± 0.22 kg/m^3^) and a reduced thickness (50.28 ± 3.52 mm), trending closer to the short fiber formulation (100S). These findings highlight the critical role of fiber morphology in determining the final foam structure, with increasing proportions of long softwood fibers consistently leading to lower density and greater thickness. This tunability enables precise tailoring of foam characteristics for target applications such as thermal insulation, cushioning, or lightweight structural components.

Fiber foam from short eucalyptus fibers (100S) showed the highest tensile and bending force and the highest elastic modulus. This is expected due to the higher density of foam, more uniform fiber network and better inter-fiber bonding typical of shorter fibers. Its strain is moderate, indicating limited elongation before breaking. Fiber foam from longer softwood fibers (100L) showed lower tensile force at break and very low elastic modulus but exhibited much higher strain at break. This indicates that long fibers provide greater flexibility and ductility but weaker bonding, likely due to bulkier structure higher content of fines leading to fewer fiber intersections and entanglements in the foam structure. Fiber foam containing 75% shorter eucalyptus fibers (75S25L) is closer to short-fiber foam behavior, exhibiting higher strength and lower strain. This ratio still maintains better ductility than pure short-fiber foam while increasing stiffness and strength compared to fiber foam from pure long fibers. Fiber foam containing 75% longer softwood fibers (25S75L) is closer to long-fiber behavior, exhibiting lower stiffness and higher strain. Some reinforcement from short fibers is evident, but the network is still relatively flexible. More balanced performance between strength and flexibility is seen in fiber foam made from 50% short and 50% long fibers (50S50L). This blend benefits from the bonding of short fibers and the flexibility of long ones.

It is evident that short eucalyptus fibers contribute to higher tensile strength and stiffness but lower ductility of foams, whereas longer softwood fibers provide higher elongation and greater flexibility. Combining both types of fibers allows tailoring of foam properties. Synergistic effects of combining eucalyptus and softwood fibers result in moderate strength, stiffness, and flexibility, seen at sample 50S50L. Directional difference (MD vs. CD) is moderate; however, CD sometimes shows slightly higher force, possibly due to the foam formation process or fiber orientation seen on cross-sectional micrographs shown in Figure 3.

Even though one would expect that fiber foams made of long fibers have better mechanical properties lower density and high porosity of the 100L sample influence the tensile strength crucially due to the lower number of hydrogen bonds as well as the higher content of fines, which can decrease the tensile strength. Although high thickness, low density, and low weight are desired when talking about protective packaging, the same properties negatively influence the tensile strength of the samples. The future research where different binders will be added into the fiber foam matrix should increase the inter-fiber connections and consequently tensile strength.

On the cross-sectional view, all samples exhibit a similar fiber distribution pattern, which can be attributed to the foam-forming process. During casting, as the foam is poured from the disperser into the mold, it compacts against the bottom filter, resulting in a higher fiber concentration at the bottom compared to the middle region of the foam. Consequently, a denser and mechanically stronger layer is formed at the bottom. In contrast, this compaction effect is less pronounced at the top of the foam. While the upper filter also contributes to some compression, the resulting densification is notably lower than at the bottom.

To better understand the overall fiber architecture, cross-sectional micrographs were obtained, providing a comprehensive view of the foam structure along its thickness. These images allowed for a clear assessment of fiber distribution across different layers and highlighted the density gradient from bottom to top. In addition, high-resolution imaging was employed to examine the local microstructure, revealing subtle details of fiber orientation and agglomeration that are not visible at lower magnification. A slight fiber orientation is observed in the machine direction (MD) on the cross-section (Figure 3), which likely stems from the inclined pouring of the suspension. This orientation is also discernible in high-resolution imaging, whereas in the cross direction (CD), fiber alignment is less apparent. Visual analysis further revealed localized fiber agglomerates, which are believed to have formed during the dispersion stage. These clusters are tentatively attributed to the water content used in the formulation; however, this remains uncertain and warrants further experiments.

In our case, PCA reveals that the first principal component (PC1) accounts for 73% of the total variance in the original variables, the second (PC2) explains 20%, while the remaining components together represent only 7%. These latter components can be disregarded, as they mostly capture noise in the data. The results are shown on Figure 4.

Figure 4a shows the position of the standardized original variables (loadings plot), while Figure 4b illustrates the location of the samples (scores plot) in the PC1–PC2 space. Variables on the left-hand side of Figure 4a—3pt-Fmax-CD, TS-E-MD, TS-E-CD, TS-Sm-MD, TS-Sm-CD, and Density—are strongly positively correlated, and therefore cluster closely together. A similar clustering is observed on the right-hand side for the group comprising TS-Eb-CD, ZP, 3pt-Dmax-MD, TS-Eb-MD, and 3pt-Dmax-CD.

From Figure 4b, it is evident that the foam sample made entirely from short fibers (100S, left side of the graph) is characterized by high values of the first group of variables. In contrast, the foam made from 100% long fibers (right side of the graph) exhibits the highest values in the second group. When projecting the five samples onto PC1, the order from left to right is as follows: 100S → 75S25L → 50S50L → 25S75L → 100L. Thus, PC1 can be considered a reliable indicator of the fiber composition in the foam (long/short fibers).

As for the second principal component (PC2, vertical axis), its interpretation is less straightforward. Among all parameters, 3pt-Fmax-MD exhibits the strongest projection onto PC2 (value = 0.5). In Figure 4a, this variable clearly deviates from the others in terms of location and also lacks any strong linear correlation with other variables.

Two parameters not previously mentioned, Mass, and Thickness, are strongly correlated (R = 0.86). Since their values vary across samples similarly to those in the second group of variables, they project similarly onto PC1. However, they also show some divergence, suggesting potentially distinct characteristics. Due to the small number of samples, it is difficult to draw definitive conclusions about the nature of this divergence.

## 5. Conclusions

Fiber foams are one of the promising sustainable materials that can be used in the pack-aging sector as a protective packaging that could be an alternative for some of the currently used materials. During the production of fiber foams, there are many steps that could influence the final fiber foam’s performance, appearance, and mechanical behavior. Type of fibers, foaming agent and foaming process, dewatering, and drying are just a few of the aspects that affect the result. The first (and regarding mechanical properties, probably the most important factor) is the selection of fibers, i.e., the proportion of softwood and hardwood fibers that are used to produce fiber foams. To analyze, determine, and better understand the mechanical behavior of fiber foams, the systematic approach of analyzing five different fiber foam samples (with different ratios of long and short fibers) was used.

The process of fiber foam production was successfully established. To determine the interdependency of different parameters, the PCA was additionally performed. Through a systematic study of five fiber foam formulations with varying ratios of short and long fibers, the production process was successfully established, and mechanical performance was evaluated. PCA confirmed that fiber composition is the primary driver of foam behavior, with the first two principal components accounting for 93% of the total variance. Contrary to expectations based on papermaking, foams made from 100% long fibers (100L) did not exhibit superior strength but instead showed the lowest density (19.0 ± 0.27 kg/m^3^), highest thickness (58.52 ± 1.50 mm), and ~30% higher air permeability, resulting in weaker bonding and reduced tensile strength. In contrast, foams made entirely of short eucalyptus fibers (100S) exhibited the highest density (28.0 ± 0.34 kg/m^3^), lowest thickness (38.82 ± 4.21 mm), and superior tensile strength and modulus, though with reduced strain at break. Blends such as 50S50L achieved more balanced performance, combining the stiffness of short fibers with the flexibility of long ones.

The presented research revealed basic properties and behavior of the fiber foams. PCA confirms that fiber composition is the primary driver of fiber foam behavior. Although long fibers contribute to volume and insulation potential, their higher fines content and reduced bonding area may weaken tensile strength. However, there are many influences that must be analyzed in the future research. The influence of additives (like starch), refining degree, quantity of foaming agent, etc., have to be determined to prepare the foam with even better properties.

## Figures and Tables

**Table 1:** Samples composition.

Sample	Short Fibers Content [%]	Long Fibers Content [%]
100S	100	0
100L	0	100
50S50L	50	50
75S25L	75	25
25S75L	25	75

**Table 2:** Morphological properties of the fibers used for foam forming.

	Length [mm]	Diameter [µm]	Curl [%]	Kink Index [1/mm]	Fibrillation [%]	Fines [%]
Short fibers	0.790	13.44	11.90	1.94	1.17	58.02
Long fibers	1.961	23.97	13.32	2.50	1.30	77.08

**Table 3:** Physical characteristics of dry cellulose foams sized 15 cm × 15 cm with five different ratios of long and short cellulose fibers.

	Mass [g]	Thickness [mm]	Density [kg/m^3^]	Air Permeability [ml/min]
100S	24.38 ± 0.50	38.82 ± 4.21	27.9 ± 0.57	1229 ± 107
100L	25.00 ± 0.36	58.52 ± 1.50	19.0 ± 0.27	1719 ± 273
50S50L	24.84 ± 0.35	54.43 ± 3.04	20.3 ± 0.28	1263 ± 168
75S25L	24.82 ± 0.25	50.28 ± 3.52	21.9 ± 0.22	1194 ± 215
25S75L	24.66 ± 0.26	55.97 ± 3.75	19.6 ± 0.21	1390 ± 191

**Table 4:** Mechanical properties of fiber foams.

	Tensile Force [N]	Tensile Strain [%]	Elastic Modulus [MPa]	Bending Force [N]
100S_MD	3.91 ± 0.53	2.01 ± 0.74	5.41 ± 1.50	6.90 ± 1.51
100L_MD	1.66 ± 0.29	7.49 ± 2.74	0.36 ± 0.17	5.77 ± 1.22
50S50L_MD	2.49 ± 0.27	3.08 ± 1.32	1.68 ± 0.60	5.92 ± 2.13
75S25L_MD	2.16 ± 0.26	2.17 ± 1.04	0.97 ± 0.05	4.27 ± 2.30
25S75L_MD	2.03 ± 0.46	4.88 ± 1.83	0.57 ± 0.03	5.64 ± 1.20
	*F (4, 24) = 32.1, p < 0.001*	*F (4, 21) = 9.5, p < 0.001*	*F (4, 24) = 46.3, p < 0.001*	*F (4, 24) = 0.93, p = 0.46*
100S_CD	4.36 ± 0.84	2.88 ± 0.93	4.04 ± 0.60	7.67 ± 1.03
100L_CD	2.44 ± 0.61	6.55 ± 1.60	0.68 ± 0.29	6.54 ± 1.74
50S50L_CD	3.13 ± 0.19	4.40 ± 0.80	1.34 ± 0.21	5.72 ± 1.57
75S25L_CD	2.96 ± 0.37	2.50 ± 0.42	1.15 ± 0.06	6.59 ± 1.25
25S75L_CD	2.77 ± 0.61	3.76 ± 1.08	0.82 ± 0.20	5.89 ± 1.27
	*F (4, 23) = 9.3, p < 0.001*	*F (4, 23) = 13.8, p < 0.001*	*F (4, 23) = 100.8, p < 0.001*	*F (4, 24) = 1.75, p = 0.17*

## Data Availability

The raw data are available from the corresponding author by request.

## Abbreviations

The following abbreviations are used in this manuscript:

PCA	Principal component analysis
SDS	Sodium dodecyl sulfate
EPS	Expanded polystyrene
EPE	Expanded polyethylene
